# Recent Advances in 3D Bioprinting: A Review of Cellulose-Based Biomaterials Ink

**DOI:** 10.3390/polym14112260

**Published:** 2022-05-31

**Authors:** Wan Nazihah Liyana Wan Jusoh, Mohd Shaiful Sajab, Peer Mohamed Abdul, Hatika Kaco

**Affiliations:** 1 Research Center for Sustainable Process Technology (CESPRO), Faculty of Engineering and Built Environment, Universiti Kebangsaan Malaysia, Bangi 43600, Selangor, Malaysia; nazihahliyana.j@gmail.com (W.N.L.W.J.); peer@ukm.edu.my (P.M.A.); 2 Department of Chemical and Process Engineering, Faculty of Engineering and Built Environment, Universiti Kebangsaan Malaysia, Bangi 43600, Selangor, Malaysia; 3 Kolej GENIUS Insan, Universiti Sains Islam Malaysia, Bandar Baru Nilai, Nilai 71800, Negeri Sembilan, Malaysia; hatikakaco@usim.edu.my

**Keywords:** 3D printing, additive manufacturing, biopolymer, cellulose, cell immobilization

## Abstract

Cellulose-based biodegradable hydrogel proves to be excellently suitable for the medical and water treatment industry based on the expressed properties such as its flexible structure and broad compatibility. Moreover, their potential to provide excellent waste management from the unutilized plant has triggered further study on the advanced biomaterial applications. To extend the use of cellulose-based hydrogel, additive manufacturing is a suitable technique for hydrogel fabrication in complex designs. Cellulose-based biomaterial ink used in 3D bioprinting can be further used for tissue engineering, drug delivery, protein study, microalgae, bacteria, and cell immobilization. This review includes a discussion on the techniques available for additive manufacturing, bio-based material, and the formation of a cellulose-based hydrogel.

## 1. Introduction

The adaptation of 3D bioprinting with cell culturing and drug delivery has shown suitable trends over the past few years. One of the benefits of using additive manufacturing, such as 3D bioprinting, is its ability to work with living materials and cells during the printing process [1]. In 3D bioprinting, biopolymer hydrogel expresses perfect criteria to imitate tissue matrix and can be produced from biomaterial [2]. Hydrogel preparation commonly requires some integration with other materials to increase efficiency and performance. Based on studies conducted by some researchers, many successful findings have been concluded for the use of hydrogel, which led to its application in the human body. Moreover, culturing algae and bacteria on the hydrogel shows an excellent characteristic of hydrogel to support the growth of living cells.

Uses of natural resources from plants and microorganisms such as cellulose, lignin, alginate, chitosan, and algae have gained some interest in 3D bioprinting. All these resources can be obtained abundantly from specific parts of plants and animals but require some treatments during the extraction process. Cellulose, extracted from plants, is an attractive biopolymer in focus throughout this review. Pretreatment steps for 3D bioprinting are less complicated, and cellulose can survive during 3D bioprinting at specific process parameters [3]. Biopolymer is the main material in medical products as it shows a safe and convenient effect that is harmless to humans and animals.

In this review, 3D bioprinting involves several techniques, such as extrusion, laser-assisted bioprinting, and inkjet, which are discussed in detail. The need for biopolymer as the biomaterial ink for 3D bioprinting and biopolymer hydrogel was discussed, particularly regarding the properties and application. Then, the recent study on 3D bioprinting for uptake and release of protein, immobilization of algae, bacteria, and cell culturing of human and animal cells in the hydrogel are being reviewed. This review includes a thorough explanation of cellulose as a biopolymer for its application in additive manufacturing, including its characteristics, extraction techniques, and size reduction process. Furthermore, we emphasized the use of cellulose-based biomaterial ink and hydrogel preparation techniques. Three-dimensional bioprinting of cellulose-based biomaterial in other specific areas such as tissue engineering and drug delivery is reviewed. Finally, the visual bibliometric network and analysis are explained in the statistic of the recent study.

## 2. 3D Bioprinting Techniques

In the early 2000s, 3D printing was initially used in the medical industry before leading to 3D bioprinting. Thomas Boland’s group invented the first bioprinter at Clemson University after using an inkjet printer for cell printing [4]. From the printing of cells, 3D bioprinting then developed into bones, organ, and tissue printing, which have comprehensive advantages in tissue engineering and organ transplant.

Most bioprinters use the same techniques as 3D printers, such as extrusion-based, inkjet, and laser-assisted. A primary step required before conducting 3D bioprinting is preparing the ink material and creating a 3D model using the software. The crucial part of 3D bioprinting is the formation of bioink made up of biocompatible material and living cells that typically need some culturing process. Bioink needs to show some criteria such as suitable viscosity, shear-thinning, and capability to be crosslinked [5]. Then, computer-aided design (CAD) is software used to model a 3D structure with internal and external details such as the size of pores [5]. The .stl file refers to standard triangle language and will be used for printing by adding the file to the 3D printing software [6]. After modeling the structure, the 3D bioprinting process can commence according to several specifications based on the required printed product.

3D bioprinting can overcome the shortage of organs and increase the possibility of organ transplants. It can produce a complex structure for medical purposes with high cell survival rates in shorter times and efficient costs [7]. The 3D bioprinting of the skin has a great possibility to be produced repeatedly with faster production [8]. The customization of specific tissues or organs is applicable with this 3D bioprinting. Direct deposition of the printing ink or cells on the targeted human body part is possible by using 3D bioprinting [9].

### 2.1. Extrusion/Liquid Deposition Modeling

Liquid deposition modeling (LDM) is a 3D printing method focusing on printing the viscous liquid made up of biomaterial. Liquid ink can be added and stored in the syringe for printing and released during the process with the help of external force. The composition and viability of the biomaterial can be maintained during the process as it is placed in a controlled temperature and pressure condition. The printing temperature and build plate temperature will influence the printing of each biomaterial. Furthermore, the printing process is controlled by specific parameters such as viscosity, the flow of fluid, shear-thinning, and gelation [10]. Other than that, the geometry of printed material is crucial for 3D printing. As the size becomes smaller with a channeled structure, the printing becomes more challenging and complicated [11]. Clogging and blockage are the main problems in the printing process, and as explained, smaller-sized print core leads to clogging.

There is a general procedure for extruding 3D bioprinting of bacterial nanocellulose (BNC) by dissolution in 1-ethyl-3-methylimidazolium acetate (EmimAC) [12]. The syringe is attached to the holder and connected to the cellulose solution, and the syringe pump will manage the syringe actuator. A needle for ink dispersion is attached to the syringe. A slight increase in the temperature of ink solution can cause dilution and ease the extrusion of bioinks. The increase in the concentration of biomaterial requires an increase in printing temperature to reduce the potential of blockage in the print head and nozzle [6].

Biomaterial must be in a printable solution state, which is in a paste-like form [13]. Additional chemicals such as isopropanol were used to clean the building plane and induce an easier transfer of printed material. For example, in extrusion bioprinting, the dual crosslinking technique is the recent finding where two different biomaterials, polyethylene glycol and cellulose nanofibrils (CNF), are required [14]. It needs to be crosslinked before and after bioprinting by placing it under the light for photo-crosslinking. The crosslinking of biomaterial improved the properties of the printed product and showed better potential to be used as a bioinks. LDM technique is suitable for printing liquid bioink due to the printer’s design.

### 2.2. Stereolithography

Stereolithography, a compatible 3D printing technique, uses digital light processing where an ultraviolet lamp is used as the curing agent [15]. Moreover, the excess resin can be eliminated from the printed material by cleaning with a suitable alcohol solution. Recently, a new printing technique was discovered in which it uses fast hydrogel stereolithography printing [16]. This technique can produce a centimeter-sized printed product such as a hydrogel scaffold in several minutes by using photopolymerization. The printing setup uses digital light processor (DLP) devices to print the image on the photosensitive material in the liquid tank. Some parameters such as the condition of photopolymerization, force-driven, and velocity of hydrogel need to be controlled. Low printing duration gives advantages to this process as it lowers the chances of cell injury and deformation due to lesser printing time.

Besides that, it uses a liquid resin that can provide natural support in printing the hollow structure. Photopolymerization is an important process that affects the product. It can be managed by controlling the energy, printing speed, polymer concentration, and coefficient of photoinitiator absorption. In addition, there are studies on applying low force stereolithography using a Form 3B printer with seven printing resins in the formation of microfluidic devices for biological application [17]. Technically, low force stereolithography uses a combination of the galvanometer, laser beam, filter, fold, and parabolic mirror in the printing process. The maximum resolution is obtained by manipulating the channel and pillar using software and adjusting the orientation of the printing angle. This improved technique resulted in increased preciseness, accuracy, and less manufacturing stress on the printed product. Culturing human endothelial cells on the printed disc shows that the disc is biocompatible for cell seeding.

### 2.3. Inkjet

Inkjet printing is divided into two techniques, which are drop on demand (DOD) and continuous inkjet (CIJ). It is a contactless printer that does not cause any damage to the printed substrate during the process [18]. An exact small amount of bioinks droplet (picolitre) is released onto the substrate or culture dish [19]. Sterilization of the printer is required before adding the ink to minimize the risk of contamination. Sterilization can be performed by exposing the printer to ultraviolet light for at least overnight, spraying the cartridge with ethanol, and incubating the bio paper [20]. For inkjet printing of cellulose-based hydrogel, it is recommended to use low viscosity ink as it will ease the printing process [10]. This printing technique is able to print biomaterial ink directly on the body through an automated or hand-held device [21]. The printing is precisely based on the topography, and it is beneficial for an uneven wound area.

For 3D bioprinting of biomaterial using alginate and CNF, the centrifugation process and withdrawal of the unused supernatant enable improvement in the dispersion of CNF [22]. The accuracy of printing and excellent dispersion is determined by the micro-sized 300 μm diameters of the printer head valve. At the same time, the flow rate depends on the pressure of dispersion, time of valve opening, and distance of dosing. Additional printing speed parameter is required in managing the distance between each printed line. Besides that, stirring can lower the potential of ink from settling. CNF has better accuracy in terms of shape than alginates as it has low shear rates, higher viscosity, and shear-thinning, but mechanical force could destroy it.

### 2.4. Laser-Assisted Bioprinting

Laser-assisted bioprinting (LAB) is a bioprinting technique that uses the laser to deposit the bioink into the substrate surfaces. It applied the laser-induced forward-transfer (LIFT) effect with the involvement of three components, such as the pulse laser source, ribbon, or target, containing the bioink and substrate to accept the printed material [23,24]. The ribbon or target should be made of a non-absorbing material and coated with a thin layer of absorbing metal for easier deposition of printed material [23]. The printing process using LAB techniques takes almost 3 h for the whole printing. Moreover, it is required to have a laminar flow cabinet and incubator to reduce the chances of contamination and better printing efficiency, respectively [24]. The laser-assisted bioprinting workstation must include specifications such as the laser wavelength, pulse duration, repetition rate, beam quality, galvanometric mirror, CAD/CAM software, and printer cartridges [25].

For this technique, the bioink is in the liquid state, and its viscosity and cell composition will determine printed cell quality [25]. Furthermore, the ink solution needs to maintain its sterilization condition and viscoelastic properties during the printing process [24]. For efficient printing, analyzing the cartridges under the microscope before printing can help maintain the concentration and uniform distribution of cells to be entirely deposited on the slide [24]. Besides that, loss of water and stable viscoelastic properties can be controlled by locating the cartridge and holder onto the ice blocks before printing. Laser-assisted bioprinting is an advanced bioprinting technique that can specify the cell density and the 3D organization of cells [23]. Other than that, it can imitate the physiological performance of the actual component. This technique enables automation in processing and high reproducibility of the printed material.

## 3. Potential and Limitation of Hydrogel as Biomaterial Ink

### 3.1. Biomaterial Ink

**Figure 1:**
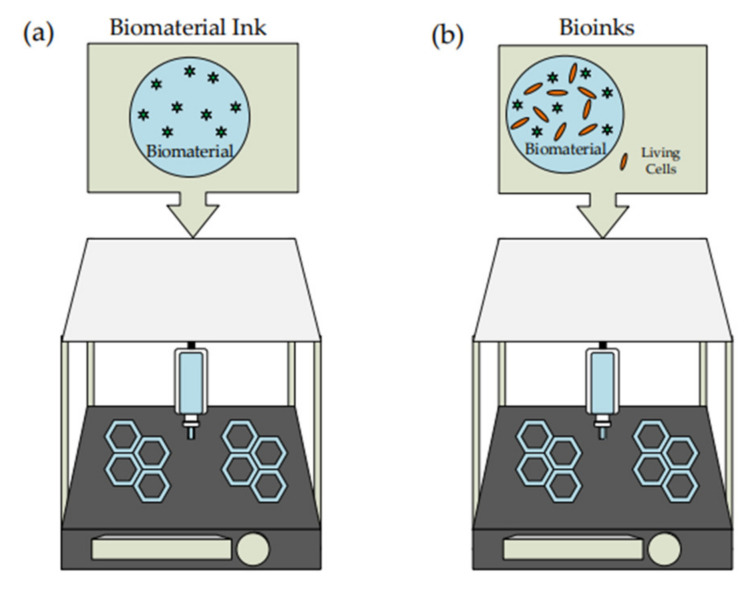
Difference between (**a**) 3D printing and (**b**) 3D bioprinting with the illustration using liquid deposition modeling (LDM) technique.

Three-dimensional bioprinting is an example of additive manufacturing. It is an advanced 3D printing technique that can print living cells using biomaterial to support and enhance the cells. The biocompatible material will undergo modification based on the desired printed living cell before being added to the syringe. By using 3D bioprinting, it avoids a high-risk cell seeding process and promotes high distribution of cells at multiple locations as well as high cell density [26]. A significant comparison between 3D printing and bioprinting was illustrated in Figure 1. Many studies have been conducted on the cell and tissue for application in the medical field.

Significant differences in 3D printing and 3D bioprinting are based on the type of ink being extruded from the nozzle. Biomaterial ink is the typical type of ink used for 3D printing involving bio-based material in ink production. The biomaterial can interact with or respond to the living cells where it is widely applied in medical treatment [27,28]. In the late 1800s, the uses of biomaterials gained much attention and are being used widely for medical purposes [29]. The interaction of biomaterial with biological cells might have good or bad effects depending on the characteristics of the biomaterial [28]. At the early stage of its development, the biomaterial is relatively inert and cannot be totally accepted by the body [27]. The biomaterial should be biocompatible, non-toxic, bioactive, and biodegradable for the human body [27,30].

For 3D bioprinting, the ink consists of a combination of living cells with the biomaterial called bioinks [31]. Generally, it can be divided into two categories in which natural and synthetic bioinks [32]. Bioink should express similar criteria as the other ink, such as excellent mechanical properties and the ability to retain the cell throughout the printing process. As it involves biologically, the bioinks must be biocompatible and cytocompatible toward the living cell and extracellular matrix [31].

### 3.2. Potential of Biopolymer Hydrogel

Biopolymers are monomers attached by a covalent bond to form a larger molecule. The phrase bio refers to the material produced by living things such as plants or microbes. For medical application, most biopolymer is derived from microorganisms and are suitable for drug delivery and tissue engineering [33]. The increase in waste production from agriculture triggered the development of biopolymer material. Moreover, it can reduce the possibility of global warming as the waste disposed into landfills decreases [34]. Some controlled conditions are required in producing biopolymer where the microorganism needs certain nutrients and a suitable environment for its development [33].

Cellulose and alginate are the common biomaterials used in preparing hydrogel for the immobilization of microalgae [35,36]. CNF as the medium for immobilization of algae and cyanobacteria shows better outcomes as it is in nanostructure with a high aspect ratio. Moreover, it is fully transparent to penetrate light sources for cell growth [35]. At the same time, alginate has suitable biocompatibility, simple handling, and can use thin layer immobilization where it can provide constant light for capturing cell structure [35,36]. Further study has been conducted on the loading and releasing of protein in BNC hydrogel using a high-speed technique [37]. It is claimed that the adsorption process increases rapidly with the high-speed approach, but the release is slower compared to the adsorption technique. This occurs due to instability of the protein, thickness of fiber, size of pores, and density.

High-speed methods produce a higher thickness of fiber that partially close the pores and a higher density that cause more obstacle in transport. However, a robust cell is required during the 3D bioprinting process to ensure that those cells can endure physical and biological stresses such as shear stress and unstable pH [9]. A study on superabsorbent hydrogel was performed using sodium carboxymethyl cellulose (NaCMC) and cellulose in the presence of NaOH/urea aqueous with epichlorohydrin (ECH) as the crosslinker [38]. It is suitable for smart swelling and controllable delivery of protein.

Besides that, the loading and release of BSA from injectable polysaccharides-based hydrogel, prepared from cation octa(γ-chloroammoniumpropyl) silsesquioxane (OCAPS), chitosan, and oxidized hydroxypropyl cellulose show a successful analysis [39]. The release of protein is analyzed using an ultraviolet spectrophotometer. The formulation for encapsulation efficiency (EE) and drug loading efficiency (LE) is mentioned in Equations (1) and (2).

(1) $$EE=\frac{m_{1}-m_{2}}{m_{1}}\times 100\%$$

(2) $$LE=\frac{m}{M}\times 100\%$$

$m_{1}$ is referring to the total mass of BSA added, $m_{2}$ is the mass of BSA in the solution, m is the total mass of BSA in the drug-loaded hydrogel, and M is the mass of hydrogel after drying. Their findings stated that the EE of hydrogel is approximately 100% and has an efficient encapsulation.

Other than that, there is a study on the photo-controlled release of BSA from photo-response hydrogel (PR-gel). It is developed from a combination of 4arm-PEG and azobenzene into CNF [40]. The release of BSA was conducted under the presence of ultraviolet light, which will cause some changes in the hydrogel structure. The suitable wavelength using UV radiation is 365 nm, while visible light is 400–500 nm. Leakage and diffusion of protein from the hydrogel structure and *trans*-to-*cis* photoisomerization are the possible causes of an increase in the release rate of protein.

The release of protein using BSA and lysozyme from sodium alginate-bamboo-bacterial cellulose hydrogel shows that the lysozyme release is faster during the early phase [41]. The release rate control by electrostatic adsorption gives suitable pH-dependent release. Meanwhile, BSA has a lower release rate because of stronger hydrophobic adsorption generated by lignin. The hydrogel used in this study has an excellent ability to support hydrophilic protein-based drugs, and it does not show any cell toxicity during the process.

Another study is on applying multi-layered spheres for protein-based drug release using alginate and cellulose nanocrystals (CNC) [42]. The first and third layers of the sphere consist of alginate, while the second layer combines CNC and bovine serum albumin (BSA). BSA was loaded into the multi-layered sphere by incubation in the gastric fluid, and the release was analyzed using a protein assay kit. It is explained that multi-layer spherical hydrogel can manage and make a sustained protein release into the gastric environment.

Bacterial nanocellulose (BNC) can be used in drug delivery to observe the loading and release behavior of protein albumin [43]. BNC for this study is being generated by *Gluconacetobacter xylinus* using bottom-up approaches by culturing the aerobic bacteria on the hydrogel. The high ability for protein absorption is analyzed on pure BNC compared to wood pulp cellulose [43]. The study concludes that BNC is the best choice of carrier for protein uptake. Despite that, some improvement needs to be made as the rate of protein uptake by hydrogel is faster than the rate of release. The loading rate of the drug depends on the time of loading and concentration of protein while released based on the amount of loaded protein. Releasing protein requires additional time compared to the loading process due to the concentration gradient, which causes a low diffusion rate. The application of BNC shows similar behavior as the other hydrogels with more environmental and user-friendly features.

Another study on protein enrichment uses superabsorbent polymer (SAPs) hydrogel for membrane technologies [44]. The protein enzyme (BSA) shows an increase in concentration through the forward osmosis process. The study explained that the protein is intact and has a low rate of denatured due to less membrane fouling, high performance of dewatering, and no issues related to reverse draw diffusion. Besides that, an increase in the contact area of the membrane, hydrogel mass, protein concentration, and number of setups in series would produce better enrichment factors. Briefly, a summary of protein immobilization for uptake and release in the hydrogel is simplified in Table 1.

Furthermore, gel entrapment is one of the most practical active immobilization techniques for algae in which the substrates are made up of biomaterial such as polymers, proteins, and polysaccharides [45]. Polyacrylamide hydrogel has the potential to be the medium for the immobilization of microalgae [46]. The sizes of pores play important roles during immobilization, which increases the pores sizes causing the microalgae to move freely. As the pore sizes reduce, the microalgae can be kept in their best state for about 20 days. Polyethylenimine (PEI) can be used as an immobilization medium for microalgae due to its biocompatibility [47]. The additional crosslinker impacts the growth of microalgae and the rate of immobilization. Epichlorohydrin shows a better attachment to microalgae compared to diethylene glycol diglycidyl ether (DGDE). This explains suitable crosslinker is required for a suitable attachment of microalgae.

Several studies have been conducted for the immobilization of bacteria on biomaterial for various applications. Physical adsorptions were used to immobilize *Bacillus velezensis* strain on the microsphere of sodium alginate (SA)/polyvinyl alcohol (PVA)/nano zinc oxide in the treatment of slaughter wastewater [48]. The higher degradation rate of chemical oxygen demand and lower growth rate of the pathogen have resulted from the application of immobilized bacteria on the combination of biomaterial. Besides that, bioluminescence bacteria can be immobilized in the alginate beads to sense explosive material [49]. Even though the detection using bacteria is successful, the exposure of engineered microbes might impact marine life.

For the removal of polycyclic aromatic hydrocarbons (PAHs), soil washing can be carried out efficiently by immobilizing the bacteria in PVA-SA hydrogel beads [50]. The removal rate is higher and faster at a medium concentration of bacteria, and the hydrogel can be reused, which reduces the cost and waste generation. The bacteria in the immobilized hydrogel show better performance on adsorption and degradable activity than unattached bacteria. Immobilization of the *Clostridium intestinale* strain on the alginate hydrogel beads produces more biological hydrogen and reduces the formation of the end product [51].

**Figure 2:**
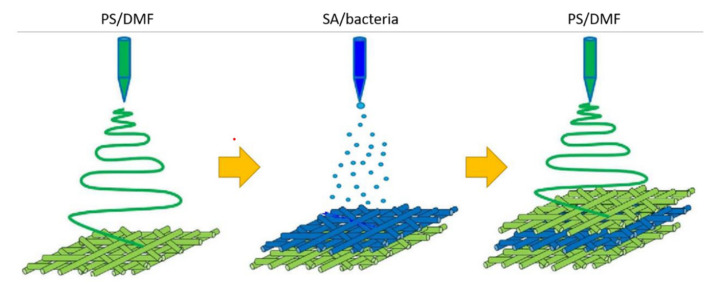
Preparation of alginate hydrogel for the immobilization of bacteria in the layer. Reproduced from [52] which is licensed under a Creative Commons Attribution-(CC BY 4.0) International License (http://creativecommons.org/licenses/by/4.0/, accessed on 17 April 2022).

Cell entrapment is a suitable method for the production of hydrogen as it is not destructed in the hydrogel and grows at a steady state. Alginate hydrogel and electrospun polystyrene nanofiber were used as the substrate for immobilizing the bacteria cell. It can provide suitable binding, cell protection, high cell activity, less restriction for cell growth, and lower toxic generation [52]. Immobilization using the layer by layer technique gives better stability to the structure, suitable thermal resistance, and lowered discharge potential of the immobilized cell. Figure 2 shows the layer by layer technique for immobilized bacteria on the hydrogel. Besides that, functional living ink (Flink) can be obtained by encapsulating the living bacteria cell in biocompatible hydrogels such as hyaluronic acid, k-carrageenan, and fumed silica [53].

## 4. 3D Bioprinting Application

**Figure 3:**
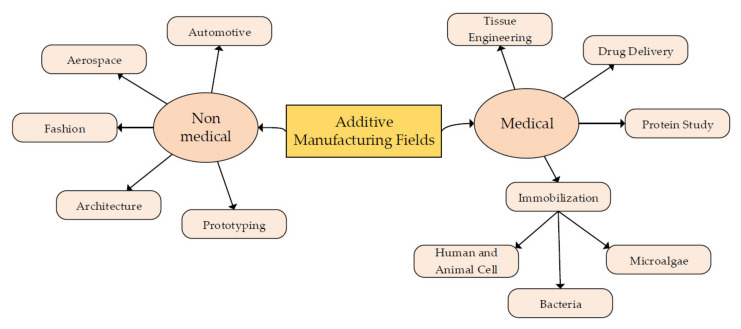
The fields involve the application of additive manufacturing for more efficient and advanced products.

Additive manufacturing has been widely used in various industries and has focused on medical applications for the past few years. Additive manufacturing in the medical field is widely known as 3D bioprinting. The significant difference between medical and non-medical fields application depends on the material used as printing ink. Three-dimensional bioprinting used biomaterial with living tissues or cells in the printing process where it required additional attention and care in handling the bioink. Applying additive manufacturing in the medical field has improved the development and technology of this industry. Tissue engineering and drug delivery are areas where 3D bioprinting is widely used. Figure 3 shows some fields and industries that started applying printing techniques to ease and faster the production process.

### 4.1. Tissue Engineering

**Figure 4:**
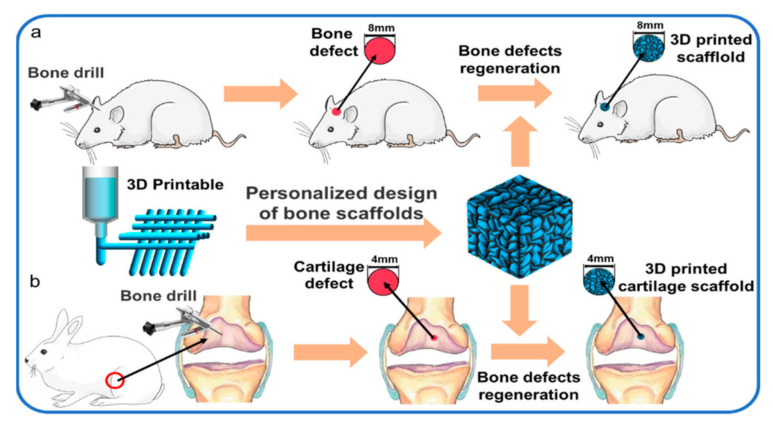
Printed bone scaffolds can replace the bone and cartilage defects in rat and rabbit, (**a**) regeneration of skull defect in rats; (**b**) articular cartilage regeneration in rabbits. Reproduced from [54] which is licensed under a Creative Commons Attribution-(CC BY 4.0) International License (http://creativecommons.org/licenses/by/4.0/, accessed on 17 April 2022).

Three-dimensional bioprinting in tissue engineering has seen rapid development. Tissue engineering refers to a field in which engineering uses living things such as cells and tissues. Biomaterial will be the primary resource in constructing new tissues and repairing damaged tissues or organs. Tissue engineering has become one of the important fields in biomedical application due to its high potential in functionalizing biomaterials and exploring various interesting medical areas. Figure 4 shows the successful application of a 3D-printed hydrogel bone scaffold for replacing the tissue in rats and rabbits. Three-dimensional bioprinting in tissue engineering should have outstanding biocompatibility and be suitable for human use without causing any rejection or harm.

Some studies have implemented 3D bioprinting in tissue engineering using other biomaterials. Inkjet bioprinting to print alginate hydrogel shows promising results and process efficiency [55]. They developed their inkjet printer using a bio clean bench to reduce contamination in printed material. The printer does not require any electrical energy during printing. It can produce a smaller printed material and understand the effect of beads size in 3D bioprinting.

Additionally, printing vascular tissue raised some concerns on selected criteria such as chemically modifying the biomaterial, controlling the size of pores, and involvement of growth factors [56]. It also stated that additional support is required during the printing of vascular tissue, such as for sacrificial ink, which can be removed upon printing. Recently, the conductive material in tissue engineering has been applied with 3D printing techniques [57]. The use of conductive material as ink can produce a more functionalized printed product that can reduce the rejection by the human body.

### 4.2. Drug Delivery

**Figure 5:**
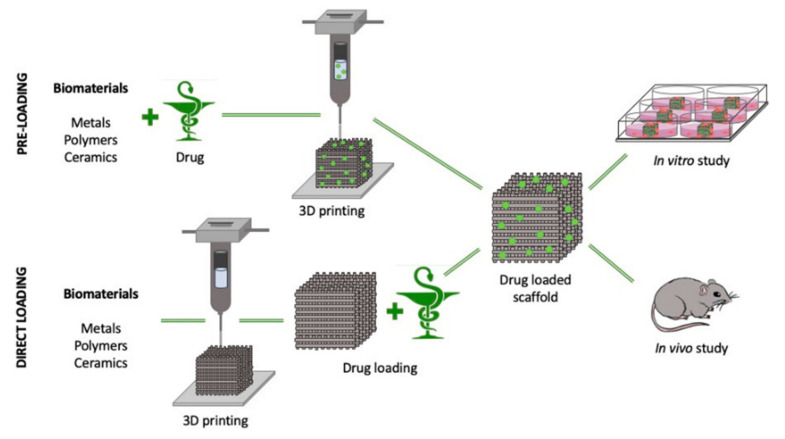
Loading of drug into the scaffold before and after printing for the in vitro and in vivo study. Reproduced from [58] which is licensed under a Creative Commons Attribution-(CC BY 4.0) International License (http://creativecommons.org/licenses/by/4.0/, accessed on 17 April 2022).

A drug delivery system commonly refers to the transport of drugs to the targeted area in a controlled manner. The specific untreated area in the body is being detected, and the drug is released based on body requirements. The loading techniques of the drug into the 3D-printed hydrogel scaffold for in vitro and in vivo study have been illustrated in Figure 5. For the pre-loading technique, the drug is introduced with the biomaterial before printing, while it is introduced once the hydrogel scaffold has been printed for direct loading [58]. Previously, many issues related to the uncontrolled release of the drug have been identified, and this situation can lead to side effects and further harm to the consumers.

Recent studies by researchers have shown that applying biomaterial and 3D bioprinting has a high possibility of encountering problems related to the drug delivery process. Three-dimensional bioprinting has been widely used for this system as it enables the modification of the drugs and gives a faster production period. There are few mechanisms for a controlled released system of drugs, such as temporal-controlled, distribution-controlled, and erosion-controlled [59]. The drug delivery system is divided into two major groups, which are oral and transdermal delivery systems. The oral drug delivery system is administered into the body by passing through the oral system, while transdermal delivery is through the skin. Each controlled release and drug delivery system has its roles and benefit for the pharmaceutical field.

Further studies on the solid drug dosage form have continued to determine the effect of geometry and composition on the dissolution of the drug [11]. The composition will highly affect the dissolution of the drug compared to its surface area and geometry. Mini tablets generated from passive diffusion provided smoother surface structure and minor surface defect compared to hot-melt extrusion as it depends on the quality and density of the filament. Hot-melt extrusion can obtain higher drug loadings and weights but lower filament homogeneity. Moreover, this also led to complexity in getting the desired filament diameter.

### 4.3. Protein Study

Protein is one of the critical nutrients abundantly present in the human body. Every cell has protein for the growth of the cell [60]. Protein is a substance made up of amino acids, a group of organic molecules. There are a few types of protein, such as collagen, gelatin, silk, polysaccharides, milk, and wool protein [61]. The application of protein is quite complex due to its three-dimensional structure and strong molecular force.

Protein expresses some unique characteristics and properties to ensure that it has the ability to work with cellulose-based hydrogel. The solubility of the protein in water depends on its structure and pH. Higher acidity and alkalinity tend to increase the solubility of the protein. Protein can react with various chemical and mineral acids to form other products. It is being tested in multiple methods such as the Biuret test and Pauly test [62,63]. A study on the loading of protein was conducted in BSA solution for three days and then released using phosphate buffer solution (PBS) [38]. The release behavior of the protein shows a biphasic release pattern in which it starts with a gradual release then followed by a slow release. It is claimed that larger-sized hydrogel pores provide faster protein release and are highly capable as the polymeric carrier for protein transport.

### 4.4. Immobilization of Microalgae

Algae is an aquatic organism that will undergo photosynthesis to produce useful material. In general, a light source will aid algae in converting water and carbon dioxide into biomass. Commonly, algae are divided into two main groups, which are macroalgae and microalgae, and have complex structures [64,65]. Size and cellular structure are the characteristics used to classify the algae into the two main groups [66]. Macroalgae, larger-sized algae, have a multicellular structure, while microalgae are unicellular cells and smaller in size [67]. Naturally, algae can be obtained from ponds, oceans, and wastewater, but for commercial production, it can be cultivated in open or closed systems [66].

Recently, the production process used a minimum cost high yield method, and biomass production can increase up to 98% using open system cultivation [68,69]. The cultivation process of microalgae is faster where it can produce twice the actual amount within 24 h [70]. Besides that, the quality and amount of light supply, irradiance, photoperiod, and seasons of the area can also impact the growth rate [71].

Because of algae’s characteristics, it has been used in various applications and fields such as the feedstock for biodiesel, source of carbon for fermentation, wastewater treatment, atmospheric carbon dioxide mitigation, and the pharmaceutical industry [66,72]. Accessible production and high yield of microalgae have gained the attention of researchers worldwide to apply algae in several potential sectors. Microalgae’s advantage is fast adapting to an unfamiliar environment and can be produced in large quantities in less time, maintenance, and cost [69]. Lipids, proteins, and carbohydrates are the main components produced from microalgae biomass in huge amounts and are the primary producers of food sources for the other aquatic organisms [72,73].

Hydrogel as the medium for immobilization of microalgae has been proven in some studies based on its ability to grow. The soft structure with high water content can support the growth and cell activity for culturing microalgae. For immobilizing microalgae, two approaches can be applied, which are the encapsulation of the microalgae in the polymer matrix and creating substrate from natural sources before seeding the microalgae [46,74]. Nanoporous silica matrix is an example of a polymer matrix, while agar, alginate, and cellulose can be used as the source to fabricate substrates such as hydrogel.

Bold basal medium (BBM), a freshwater algae medium, is an important medium for growing algae. The ability to immobilize microalgae can be understood by inoculating the hydrogel or agar with the microalgae species and then further culturing with BBM [46]. Another method can be performed by preparing agar with BBM followed by inoculating microalgae on the top of the agar surface [75].

### 4.5. Immobilization of Bacteria

Bacteria, the most simple and abundant organism with more than 5000 species that have been identified, hold a critical function in supporting the ecosystem and the environment [76]. The classification of bacteria depends mainly on the metabolic and genetic characteristics as their structure does not differ much from each other.

A few factors affect the growth of bacteria, such as pH, temperature, nutrients, oxygen level, concentration, and species of bacteria [77]. Bacteria have been applied in many industries due to their potential and properties such as biological hydrogen, wastewater treatment, soil washing, biofilms, and biosensors [50,51,78,79,80]. Immobilization of bacteria on the surfaces depends on the characteristic of the material, the bond between surface and linker, physiological medium, and properties of bacteria [80]. Bacteria can be immobilized in two different techniques, which are through attachment to hard surfaces and hydrogel. Adsorption, crosslinking, cell entrapment, and cell encapsulation are the common techniques applied in the immobilization process.

In the immobilization of bacterial cells, 3D printing has the potential to be a new technique for encapsulating cells in less processing time. Multiphoton lithography, one of the laser printing methods, has been used to encapsulate bacteria cells within the BSA hydrogel [81]. Chemical crosslinking of the polypeptides requires gelatin from BSA and photo-sensing molecules at a suitable temperature. Bacteria will then be encapsulated in the thermal control BSA hydrogel and starts to grow within the 3D structure. Bacteria can grow rapidly in the culture medium without showing any potential for cell death because of exposure to extracellular factors. The micro-3D printing technique can be used as the most potent approach in rapidly producing an abundant amount of complex organisms with precise dimension and resolution. Despite this, it requires more costly equipment and is less suitable for a high production rate.

Multimaterial direct ink writing technique was used in fabricating the hydrogel digitally, and it gives unlimited choices of shapes and compositions. The bacteria can grow freely in the printed structure, form any desired shapes, and have a higher survival rate. The size of the hydrogel will increase up to 1.5 times larger than the actual size after immersion in the bacterial medium due to some deviation in the concentration of ions between hydrogel and culture medium.

### 4.6. Immobilization of Human and Animal Cells

Recently, 3D printing has changed its direction toward the medical field, especially for human applications. Many studies were performed involving human and animal cell tissues. The characteristics and properties of cell tissues call for extensive attention as it requires an appropriate working environment and handling procedure for cell survival. The growth of cell tissues need compatible surrounding with enough supply of nutrient, and hydrogel is one of the choices suitable to mimic the body tissue and provides all necessities. The application of hydrogel and 3D bioprinting will be thoroughly discussed in this section.

The hydrogel is made up of PVA, SA, and MXene without the presence of any chemical crosslinked [82]. MXene is the transition metal carbide capable of incubation and can improve the conductivity properties of the hydrogel. The conductivity of the hydrogel is analyzed by attaching the LED sensor to the hydrogel. It is found that the LED sensor increases in brightness once the hydrogel is compressed, and the response time is slower. This hydrogel can detect motion responses, such as at the knee or wrist, by converting the movement signal into an electrical signal. The sensor can be located directly on the human body for reading and analysis. These findings show that hydrogel has suitable conductivity and works well for sensors based on the stress exerted on its structure.

A study has been conducted using phenol-grafted polyglucuronic acid (PGU) as bioinks for 3D bioprinting [83]. The ability of the hydrogel to be used in cell culture was analyzed using mouse fibroblast and human hepatoma cells. PGU is one of the potential microbial polysaccharides for bioprinting ink as it requires less pre- and post-processing. The gelation of PGU-Ph solution required horseradish peroxidase (HRP)-catalyzed reaction and hydrogen peroxide. A faster gelation time is observed with the increases in the concentration of PGU-Ph and decreases in the concentration of hydrogen peroxide. Cell viability of mouse fibroblast and human hepatoma cells in PGU-Ph hydrogel shows high viability percentage of 95% and 94%, respectively, on day 2 and kept constant until day 11. Mouse fibroblast does not form any cell aggregates, while human hepatoma cells aggregation kept increasing during the culture process, which means that the cell kept growing in the hydrogel.

Recently, there have been a few studies on the involvement of 3D bioprinting in scar therapies. Besides that, printing hamster cells using a thermal inkjet is successful as it is represented by green fluorescent during the viability study [20]. Cell printing can be used as one of the methods of delivery to the targeted cell. It is stated that the cell is in suitable condition and shape during and after the printing process. The formation of transient pores can automatically fix the defect and produce a suitable transfection efficiency.

A model on human hypertrophic scars was constructed by producing bioink from decellularized extracellular matrix and hydrogel from alginate and gelatin [84]. Human hypertrophic scar cells show potential characteristics for drug testing and exhibit the same profile as scar at a specific gene and protein level. Preformed cellular aggregates (PCA) are the bioprinting technique used to form a scars model that can help in tissue morphogenesis. It is suitable for spontaneous multicellular processes and can reduce modeling time. The viability of the cell is considered high, which is more than 90%. Moreover, it does not express any changes in cell morphology. However, it increases the rate of proliferation and the ability to migrate. This study successfully explains the formation of scars by using hydrogel and a 3D bioprinting technique that can mimic the native scars and be applied in drug delivery.

Other than that, there is a study on reducing the potential of scar contraction using a dermal extracellular matrix (dECM) in a 3D-printed dermal analog [85]. Bioink dermal extracellular matrix powder (dECMp) is prepared from dECM with the same material composition and morphological characteristics. Crosslinked printed dermal analogs (PDA) show a great cytocompatibility (>75%) through the analysis with L929 fibroblast compared to the control medium. It can minimize the contraction of the wound and the formation of scars. The crosslinking used is glutaraldehyde (GA) solution, and its concentration will control the printed shape and strengthen the tensile properties. Hydrogel in scars therapies shows an excellent characteristic for application with drugs and lowers the contraction of scars.

Moreover, there is some study on microparticles and beads for better efficiency as the size is reduced and the surface area increases. Alginates microparticles can be fabricated using multichannel 3D printing for protein encapsulation. They are suitable for human mesenchymal stem cells once combined with a collagen scaffold [86]. The printing devices were built with more than one channel for ink deposition based on the phases, and it uses droplet-based techniques. Shear stress is the factor that forms microparticles with spherical shapes, while the size of microparticles is affected by the flow rate of mineral oil and alginate solution. The microparticles were used to study the release of BSA, and it shows the same pattern, which is the burst release for the first five hours and then continued with a fast release. Burst release should be controlled as it can concentrate protein in one place and lower the performance of protein encapsulation. Human mesenchymal stem cells show suitable viability after five days in collagen scaffolds with alginate microparticles, as more than 90% of cells can be analyzed from a cell staining kit. Furthermore, the microstructure of alginates does not affect the morphology and spread potential of cells.

Another study uses hydrogel granules from the extrusion of hyaluronic acid hydrogel through porous nylon fabric in assisting human-induced pluripotent stem cell (hiPSC)-derived neural networks [87]. Three-dimensionally printed tools able to reduce time and produce repetitive hydrogel granules for the extrusion process. Cell viability in granules hydrogel is better than in bulk hydrogel, and it can encourage cell attachment and extension. Granules have an excellent ability to match any tissue type due to boundary intersection. Moreover, the size of granules hydrogel will influence its performance as the smaller size provides more active space than larger granules. In vitro cell culture treatment of neurology disease and drug screening can be performed efficiently by applying the multiphase cell encapsulation of hydrogel.

## 5. Cellulose-Based as Biomaterial Ink

### 5.1. Preparation for Biomaterial Ink

Cellulose-based biomaterials express great potential in 3D bioprinting. CMC can be used as bioinks due to its excellent shear adjustment, alteration of viscosity, and attribute of shape [88]. For example, the application of direct ink writing as the printing technique for water-based ink of NaCMC with humidity control [89]. Suitable print head and nozzle humidity are crucial in printing for robustness and repeatability of the process. Moreover, it can avoid faster drying of the ink bridge and ensure a higher quality of printed surface as the humid surrounding might increase the water content.

**Figure 6:**
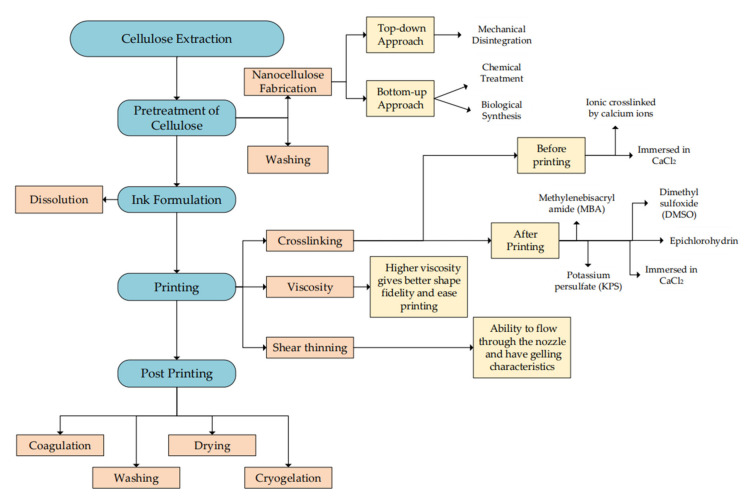
Preparation techniques in producing cellulose and nanocellulose by top-down and bottom-up approaches involve mechanical disintegration, chemical treatment, and biological synthesis.

Whereas nanocellulose has great potential to be printed due to its ability to pass through a micro-sized nozzle and release a precise shape. Nanocellulose is a natural fiber extracted from cellulose with a size less than 100 nm in diameter and has up to micrometers in length [90]. The preparation of nanocellulose is divided into three effective techniques, which are mechanical disintegration, chemical reaction, and biological reaction [91]. A brief explanation of the common techniques applied is listed in Figure 6. The type of nanocellulose is determined by the method of extraction. Mechanical disintegration results in nanofibrillated cellulose while extraction using chemicals produces CNC, and biological reaction is used to produce BNC [3].

Mechanical disintegration is used to break down cellulose pulp into smaller sizes and commonly uses fibril delamination to improve the mechanical properties of extracted nanocellulose. The standard mechanical treatment is ball milling, blending, ultrasonic, and electrospinning. The mechanical technique has its disadvantages where high energy is required for the process and can lead to a low amount and quality of the product [92]. High-pressure homogenization is one of the efficient processes. A tiny nozzle will deliver the cellulose slurry at high pressure and change into nanocellulose due to impact and shear force [92]. Because of the very small nozzle size, clogging might be the main problem, and it can be controlled by slightly reducing the size of cellulose prior to the process.

Other than that, microfluidization requires a pump to supply pressure and a chamber as a place for the shear process to occur [92]. Besides that, ultrasonication is a process that uses sound energy to cause agitation of particles. It applies an extreme oscillation using the hydrodynamic force of ultrasound [93]. Another technique under mechanical disintegration is grinding and cryocrushing. The grinding process has two grindstones, which are static and rotating grinding stones. The slurry will flow in between the two stones to form nanosized fiber [94]. Cryocrushing involves immersing cellulosic fiber in liquid nitrogen and turning it into small pieces by high pressure from mortar and pestle.

Besides that, the chemical treatment can also produce nanocellulose, and some possible techniques are acid hydrolysis, carboxylation, sulphonation, and ionic liquid. Extracting cellulose using ionic liquid is environmentally friendly as most of the solvent can be recovered. The main and commonly used technique for chemical treatment is acid hydrolysis [90]. The ordered region will undergo a hydrolyzation process using acid, while the disordered will remain without any treatment. It involves chemicals such as sodium hydroxide, sulfuric acid, and sodium bicarbonate [3]. The suspension is centrifuged and decanted with deionized water a few times until it obtains a neutral pH.

In the biological approach, enzymatic hydrolysis can be applied for pretreatment due to its potential to produce less chemical waste and energy consumption. Enzymatic hydrolysis uses the application of enzymes in digesting and transforming the structure of cellulose, and typically, this process takes a longer time [90]. Less adjustment on structure, excellent crystallinity, and thermal stability are expected when the cellulose undergoes a suitable pretreatment before the extraction [95].

### 5.2. Curing of 3D-Printed Cellulose Hydrogel

Hydrogels are a set of polymer chains bonded with a covalent or physical bond and can form a structure like mesh by applying a crosslinker [96]. Hydrogel is a gel-like structure, and it is water-resistant [97]. Some hydrogels are known as intelligent hydrogels as they can be temperature-sensitive, pH-sensitive, glucose-sensitive, stimuli-sensitive, protein-sensitive, and superabsorbent due to their unique characteristics [96,98]. The hydrogel can maintain its three-dimensional structure as it is a water-swollen polymer and contains a hydrophilic functional group in its structure. Due to the wet and soft structure of hydrogel, relatively low friction is required. Moreover, the hydrogels can self-heal based on their electrostatic attraction [97]. They will not dissolve in any solution and hold a high amount of water or any biological fluid [99].

There are two categories of hydrogel based on the type of crosslinker, which is physical hydrogel, where the formation is reversible and chemical hydrogel for permanent formation [100,101]. Physical hydrogel is produced from a non-covalent crosslinker, while chemical hydrogel is produced from covalent bonding. The presence of crosslinkers will affect the physical properties of the polymer and hydrogel. Table 2 describes the example of the crosslinking process that will affect hydrogel properties.

Natural hydrogel is gaining more attention for its application in the medical field [98]. There are a few types of natural hydrogel from alginate, cellulose, chitosan, and protein. The hydrogel of crosslinked alginate and gelatin is better due to its low stiffness, fast degradation reaction, excellent metabolic activity, and suitable adhesive properties compared to the unlinked alginate. Chitosan has gained recognition in drug delivery due to its smart delivery and properties that are high in biocompatibility and biodegradability. Moreover, it enables injection, direct formation of gel, and regeneration of tissue. For protein-based hydrogel, it expresses biological function with high compatibility. Suitable strength, shear-thinning, and the potential to respond to a stimulus are some other advantages of using protein-based hydrogel. Protein-sensitive hydrogel might be helpful in the advanced therapeutics field [96].

**Figure 7:**
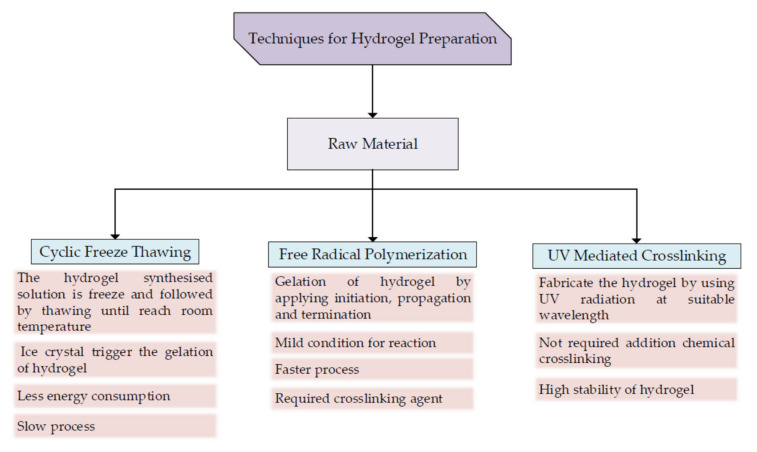
Few techniques for hydrogel formation.

Hydrogel can be produced by using a few techniques such as photopolymerization and radiation with the application of physical or chemical crosslinking [74]. For example, CNC-based hydrogel can be prepared from various methods such as homogenization, cyclic freeze-thaw processing, free radical polymerization, and UV/ion-mediated crosslinking [107]. Forming hydrogel using ultraviolet light in polymerization techniques can control the behavior of reactions and produce suitable quality hydrogel in less time [46]. The summary of common methods available in hydrogel fabrication is summarized in Figure 7.

Preparation of hydrogel from polyethylene glycol and cellulose nanofibers requires mixing of solution, sonication, and storage in a dark place [14]. Then, it will be added with calcium chloride before being placed in the mold for 3 min under blue light. Moreover, the homogenization process can be applied as the final process in extracting cellulose to form a cellulose-based hydrogel. A high-pressure cell homogenizer with the circulation of cooling water can reduce the potential of damage generated from excess heat released from the process [108].

A freeze-thaw method is another example of hydrogel production. An insoluble gel is produced in the early phase of the phases separation process and will generate ice crystals [101]. The ice crystal generated from the freezing process will trigger gelation of the solution, where it tends to be a crosslinker point [99]. A porous matrix is formed in a freeze-thaw method as the poor region of polymer will be surrounded by a rich gel region. The freezing process can be conducted at −18 ℃ while thawing at room temperature [99]. The cycle for freezing and thawing, temperature, and duration are the critical parameters in the hydrogel formation [101,109]. Gelling of CNC using the freeze-thaw method can be carried out in the water and polar organic solvent, which produces hydrogel and organogel, respectively [110]. Even though this method consumes more time, it has become one of the standard methods as it requires less energy and is environmentally friendly [101].

Another technique for hydrogel production is free radical polymerization, which has a faster rate of polymerization and can be used in an aqueous solution [101]. The crucial step for this method is the application of the initiator in polymerizing the monomer. Moreover, there are possibilities for fabricating hydrogel using radiation-induced crosslinking under mild conditions without the presence of toxins from the crosslinking agent. The production of CNC-poly(acrylamide) (PAM) hydrogel can apply this method [106]. Acrylamide was used in the free radical polymerization with the presence of some solid reagent, followed by deoxygenation using bubbles of nitrogen and stirring before adding to the mold.

Ultraviolet radiation can be used as one of the techniques for producing cellulose hydrogel. As an example, UV radiation in aqueous media was used for the formation of poly (N, N-dimethylacrylamide) (PDMA)/cellulose hydrogel [111]. The PDMA is attached to the CNC surface with the help of UV radiation in the water-soluble photoinitiator. This method does not need any extra chemical crosslinker. The interaction between PDMA and CNC polymer is a reversible physical interaction and has a suitable stability of the covalent bond. Other than that, the fabrication of CNC hydrogel uses the exposure of UV radiation under 365 nm (wavelength) [112]. For further analysis, the gel requires repeated purification to remove the excess material and freeze-drying using nitrogen for a better result.

### 5.3. Recent Trends of 3D Bioprinting Cellulose

**Figure 8:**
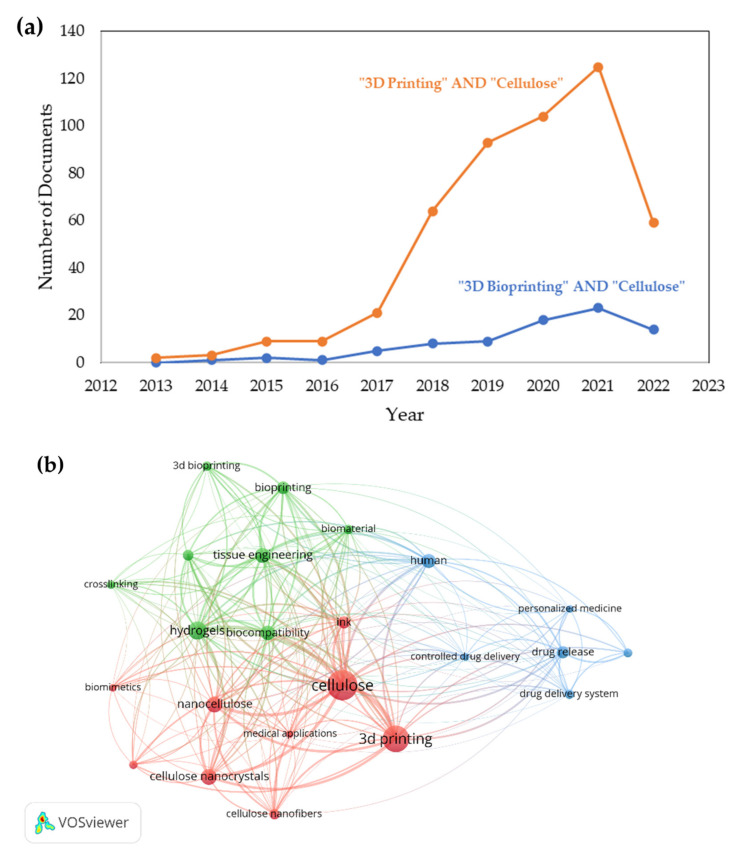
(**a**) Publication of article by year (Source: www.scopus.com, accessed on 17 April 2022) and (**b**) the co-occurrences analysis by all keywords.

All the published articles related to cellulose, 3D printing, and 3D bioprinting from Scopus were analyzed using VOSviewer for the bibliometric analysis and visualization research trends for the past 10 years. Based on Scopus, the analysis of the publication trends related to the keywords is increasing rapidly. Figure 8a shows the publication trend generated from Scopus. The number of documents only being specified to the articles type documents. The trends show that 3D printing of cellulose has a significant increase over the past 5 years compared to the 3D bioprinting of cellulose. The gap between these two keywords is huge due to the involvement of 3D bioprinting with cellulose is still new and less research. The co-occurrence analysis by VOS viewer is specified to 20 minimum number of occurrences of keywords. The generated cluster shows 3 clusters with 103 thresholds. Figure 8b shows the visualization of co-occurrences analysis by VOSviewer. Cellulose and 3D printing show the highest repetition represented by the size of the sphere, while 3D bioprinting is smaller. This can be related to the trend in Figure 8b.

Recently, there has been some implementation of 3D bioprinting with nanomaterials to study drug delivery systems. The filament used in fused deposition modeling is melted using hot-melt extrusion and then soaked in nanocellulosic solution to observe the loading process [113]. It is mentioned that the infill percentage only affects the mean weight of printed tablets. The weight decreases once the infill percentage becomes lower. A comparison of 50% and 100% infill percentages indicate that the printed material’s diameter and height are unaffected. Furthermore, choices of polymer are important in determining the drug content and the percentage of drug loading. The drug loading can be more advanced when the drug in a solid form is produced from the transformation of nanocapsules liquid suspension.

Drug delivery of 5-Fluorouracil using 3D-printed cellulose macrofibrils/calcium carbonate has shown an efficient control of drug release [114]. The cellulose macrofibrils were printed using 3D printing techniques and mixed with calcium carbonate at different ratios. The adsorption of the drug by the 3D-printed sample can be controlled by the presence of CaCO_3_. Cellulose macrofibrils can control the possibilities of rapid release. Besides that, laminating nanocellulose with calcium carbonate also shows an excellent adsorption and desorption process [13]. There is a significant difference in the percentage of adsorption and release of drugs for both nanocellulose and calcium carbonate. The adsorption of calcium carbonate is 75% higher in a shorter time.

For 3D protein printing, an early model printer has been developed using some components from the HP660C printer [115]. The printer is being modified and specified according to the requirement for protein printing, and software was used to control the location of printing. Ink cartridges used for protein printing are the original printer cartridges with multiple cleaning processes to avoid contamination. The study found that 25% of cells died after incubating for three days due to cell dehydration.

The application of 3D bioprinting produces a very neat and suitable structure for immobilization, which can affect the distribution of light and nutrients [36]. Cultivation of microalgae on bacterial cellulose is expected to merge the properties of cellulose with the photosynthetic criteria of microalgae [116]. The study found that microalgae have a huge potential for 3D bioprinting as they can be printed in layers with multiple geometries and sizes. The printed microalgae have stable physical properties as they can handle physical changes and high water content in the surrounding.

Despite the advantages, this technique might affect the physiological state of the cell. A study has been performed on fabricating hydrogel from alginate and methylcellulose to immobilize *Chlamydomonas reinhardtii* [117]. The immobilization of microalgae was observed by the increase in cell number due to the growth of cells in hydrogel under a photosynthetic environment. Moreover, the release of oxygen indicates a suitable photosynthesis process during immobilization and cell growth. Furthermore, the fabrication and crosslinking of the hydrogel are not affected by the existence of the microalgae. Methylcellulose will ease the printing process and is released after crosslinking. Releasing it will create micropores as the growth spot for algae.

Hydrogel produced from alginate and nanocellulose gives some insight into the advancement in hydrogel properties. A combination of alginate and CMC for wound dressing application can have better ink for printing due to its ability to form gelation and the suitable properties of viscosity and elasticity [118]. Moreover, the combination can lower the potential of syneresis and provide better mechanical and compression properties for resistance against deformation [119]. Fast gelation of the hydrogel can be performed with the presence of Ca^2+^ from the carboxyl group in alginates and CMC [120]. A mixture of alginates and CMC can provide better printing due to the higher viscosity of the ink solution. In tissue engineering, the combination of polyethylene glycol polymer with TEMPO-oxidized nanocellulose fiber found that the crosslinked material has excellent elastic recovery [14]. It can restore its original shape once being released from the extrusion. The composition of the ink causes variations in mechanical properties, swelling properties, and fractionation of hydrogel.

Other than that, 3D bioprinting in producing human chondrocytes has been studied using a combination of nanocellulose and alginate as the biomaterial [22]. The viability of the cell is compared between a printed and non-printed material. It is found that there is no change in the cell viability on the first day, but it starts to increase viability after seven days of the cell being cultured. Moreover, this combination of bioink is highly compatible with 3D bioprinting. Three-dimensional bioprinting can improve the tissue engineering fields by reducing the time consumed and providing a great product. The acceptance of printed material by the human body has increased with the use of hydrogel and high biocompatibility biomaterial in the printing process.

## 6. Conclusions

In conclusion, the fabrication of cellulose-based 3D-printed products has been extensively explored. In contrast, cellulose-based hydrogel shows great properties such as suitable tensile strength, elasticity, viscosity, and is lightweight. In the future, it is expected to be extensively applied in the medical and pharmaceutical industry as it has suitable criteria for biological application, and many recent studies are directed toward 3D bioprinting. Moreover, the direction of additive manufacturing starts to lead toward smart materials that respond to certain stimuli or parameters. Besides that, the application of hydrogel for the immobilization and growth of microalgae, bacteria, human and animal cells, and the properties to imitate the extracellular matrix makes it possible for any biological study.

## Figures and Tables

**Table 1:** Review on the use of protein albumin with biomaterial-based hydrogel.

Type of Protein	Hydrogel	Release and Uptake	Refs.
**Protein albumin**	Bacterial nanocellulose, BNC	Adsorption technique has slow uptake and faster release. High-speed technique has faster uptake and slow release.	[37,43]
**BSA**	Superabsorbent polymer hydrogel: Carboxymethyl cellulose, CMC, and cellulose (Crosslinker: NaOH/urea aqueous with epichlorohydrin (ECH))	Release behavior is a biphasic release pattern that is gradually and controlled by the hydrogel. Release time is controlled by the content of CMC.	[38]
**BSA**	Injectable polysaccharides-based hydrogel: cation octa (γ-chloroammoniumpropyl) silsesquioxane (OCAPS), chitosan, and oxidized hydroxypropyl cellulose	The encapsulation efficiency of hydrogel is approximately 100%.	[39]
**BSA**	Photo-response hydrogel (PR-gel): 4arm-PEG and azobenzene into CNF	Photo-controlled released BSA using UV and visible light UV shows faster release. Visible light shows slow release. Increased released rate of protein: Albumin outflow and diffusion from the hydrogel Burst release can keep dosage in its effective range.	[40]
**BSA**	Multi-layered sphere using alginate and CNC	Sustained release of the protein into the gastric environment.	[42]
**BSA**	Sodium alginate-bamboo-bacterial cellulose hydrogel	Lower released rate due to stronger hydrophobic adsorption generated by lignin.	[41]
**Lysozyme**	Sodium alginate-bamboo-bacterial cellulose hydrogel	Faster released at the early phase (conducted by electrostatic adsorption) and suitable pH-dependent released.	[41]

**Table 2:** Review on the effect and application of crosslinking toward hydrogel formation.

Crosslinker Material	Hydrogel Composition	Processing Technique	Benefit	Refs.
**CaCl_2_**	CNF and alginates	Adding CaCl_2_ to the bioink solution.	Increase viscosity of ink for suitable shape fidelity. Low tendency in shape deformation.	[22]
	CMC/SA/chitosan	Soak the film in the CaCl_2_ for 2 min.	Can improve tensile strength until certain limits.	[102]
	Alginic acid sodium salt and methylcellulose	Immersion in crosslinking solution for 10 min.		[36]
**Ca^2+^**	CNF	Ionic crosslinking before printing.	Improve viscosity of ink. Stable printed material before photo-crosslinked. Suitable shape fidelity of PEG-CNFs hydrogel.	[14]
	CNF, alginates, and colloidal lignin particle nanocomposites scaffold	Stored in Dulbecco’s phosphate buffer solution (DPBS) for 7 days with Ca^2+^ and Mg^2+^ ions.	Suitable shape stability. High swelling ratio.	[103]
	PVA and SA	1st: Crosslinked PVA by freeze-thawing method 2nd: Crosslinked sodium alginates by immersed in CaCl_2_.	Faster hydrogel response. Suitable mechanical properties.	[82]
**ECH**	CMC and cellulose	Addition of crosslinker into hydrogel solution at 30 °C and stir for 2 h. Kept for 12 h at 60 °C for the formation of the gel. Hydroxyl group of cellulose and CMC crosslinked by nucleophilic attack.	Commonly used as crosslinking for carbohydrates. Improve swelling ratio.	[38]
**Disulfide bonds**	Hyaluronic acid/carboxymethyl cellulose-based hydrogels (HA/CMC)	Oxidation reaction of dissolved oxygen in solution between the thiol groups a 37 °C.	Better structure of hydrogel. Improve swelling ratio.	[104]
**Thiol–ene photoreaction of norbornene groups**	PEG and CNF	Under visible light by exposing the blue light for 3 min at 460 nm and 25 mW cm^−1^.	Better mechanical properties of hydrogels. Enhanced stability. Stable for cell incubation in 2 weeks.	[14]
**Dimethyl sulfoxide (DMSO) and N, N’-Methylenebisacrylamide (MBA)**	Cotton cellulose	Immersion in the crosslinked solution after printing.	Provides better ability for reswelling and compression properties.	[105]
**N,N′-methylenebisacrylamide (MBA) and potassium persulfate (KPS)**	CNC−poly(acrylamide)	Stir hydrogel and crosslinker in a nitrogen bubble for deoxygenation in 10 min.	Fix the topological network. Suitable viscoelastic characteristic of hydrogel.	[106]

## Data Availability

Not applicable.
